# Ligand-based pharmacophore model for the discovery of novel CXCR2 antagonists as anti-cancer metastatic agents

**DOI:** 10.1098/rsos.180176

**Published:** 2018-07-04

**Authors:** Jinxin Che, Zhilong Wang, Haichao Sheng, Feng Huang, Xiaowu Dong, Youhong Hu, Xin Xie, Yongzhou Hu

**Affiliations:** 1 ZJU-ENS Joint Laboratory of Medicinal Chemistry, College of Pharmaceutical Sciences, Zhejiang University, Hangzhou, People's Republic of China; 2 Chinese Academy of Sciences, State Key Laboratory of Drug Research, the National Center for Drug Screening, Shanghai Institute of Materia Medica, Shanghai, People's Republic of China; 3 Chinese Academy of Sciences, CAS Key Laboratory of Receptor Research, the National Center for Drug Screening, Shanghai Institute of Materia Medica, Shanghai, People's Republic of China

**Keywords:** pharmacophore model, CXCR2, antagonists, anti-cancer metastasis

## Abstract

Metastatic cancer is considered a fatal progression of cancer worldwide. It has been shown that a key player in this scenario is the CXC chemokine receptor 2 (CXCR2). To identify novel CXCR2 antagonists, a pharmacophore model was built with the HipHop program by screening a database containing compounds which were designed based on the known structure–activity relationship (SAR) of the diarylurea series CXCR2 antagonists. Compound **1a** bearing the novel skeleton was selected from database screening and subjected to the *in vitro* biological test which showed a moderate CXCR2 antagonist potential. With further modification and exploration of SAR, compound **1e** demonstrated improved CXCR2 antagonist activity with an IC_50_ value of 14.8 µM. Furthermore, wound healing assay using the NCI-H1299 cell line indicated that **1e** showed an excellent anti-cancer metastatic effect (72% inhibition in cell migration at 50 µg ml^−1^).

## 1. Introduction

**Figure 1.**
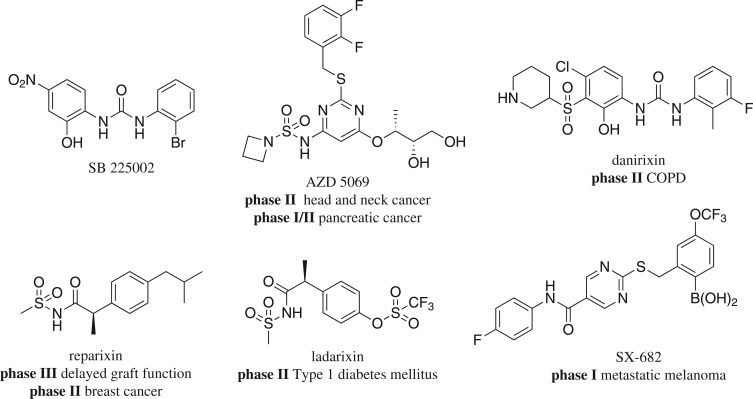
Structures of SB225002 and compounds in clinical trials.

Tumour metastasis has become a fatal disease progress which greatly influences the diagnosis, treatment and prognosis of cancer patients [1]. Generally, the metastatic progression can be simplified into three main processes: the cancer cells leave the original ‘tumour home’, travel through a ‘vessel highway’ and settle down in a new ‘tissue house’ [2]. Many inhibitors, including vascular endothelial growth factor receptor inhibitors [3,4], integrin inhibitors [5,6] or matrix metalloprotease (MMP) inhibitors [7], have been developed for the treatment of cancer cell metastasis. Recently, growing interest has been shown for the CXC chemokine receptor 2 (CXCR2), owing to their involvement in metastasis physiology [8,9]. CXCR2 belongs to the G protein-coupled receptor family, which is a seven-transmembrane protein and can be activated by several ELR+ CXC chemokines, including interleukin-8 (IL-8 or CXCL8), growth-related oncogenes (GROα, β and γ), neutrophil-activating peptide and granulocyte chemotactic protein-2 [10]. Once CXCR2 is activated by upstream ELR+ CXC chemokines, it can cause the activation of downstream signals, such as serine/threonine kinases and tyrosine kinases [8], which will lead to angiogenesis, cell metastasis and apoptosis. Its essential role in influencing tumour microenvironment makes CXCR2 an important target for anti-tumour metastasis treatment [11–13]. The first non-peptide CXCR2 antagonist **SB225002** was discovered in the mid-1990s by the GSK Company [14]. To date, five drug candidates are in clinical trials, including **Danirixin**, **AZD5069**, **Reparixin**, **Ladarixin** and **SX-682**, as shown in figure 1. In 2015, the clinical indications of **AZD5069** were expanded to metastatic head and neck cancer and metastatic pancreatic cancer, in combination with **MEDI4736** (a PDL-1 inhibitor). **Reparixin**, combined with paclitaxel, has been also used for the treatment of metastatic breast cancer since 2016. In addition, **SX-682** is in phase I clinical trial for the treatment of metastatic melanoma, in combination with pembrolizumab.

As an excellent computational tool, the pharmacophore model has been rapidly developed for the identification of novel hit/lead compounds for various targets [15–19], especially for proteins without crystal structures [20,21]. An extensive effort has been made in the attempt to apply the pharmacophore model to identify novel VEGFR inhibitors [22], MMP inhibitors [23,24] as anti-cancer metastasis agents, revealing that the ligand-based pharmacophore model is the best approach when the target geometry is not available, or the binding mode is not elucidated.

**Figure 2.**
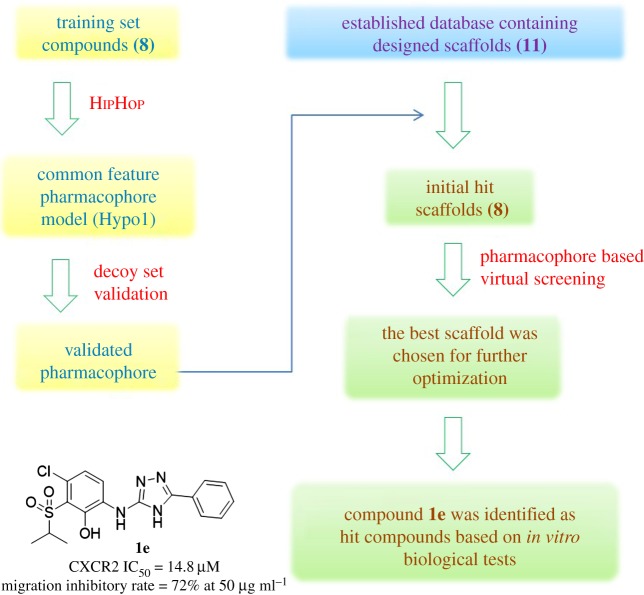
Virtual screening flow chart: pharmacophore establishment and validation; virtual screening of the mini-database; optimization of the best scaffold; and *in vitro* biological test.

At present, the crystal structure of CXCR2 has not been fully discovered; however, Neamati and co-workers have been trying to establish a ligand-based pharmacophore model which could be applied for virtual screening to find novel scaffolds of the CXCR2 antagonist [25]. Although a bunch of CXCR2 antagonists have been identified, the anti-tumour metastasis effect of CXCR2 antagonists has not been considered in most cases. In fact, we also have demonstrated the application of pharmacophores in finding various targets inhibitors [15–18], which provide a reliable tool in drug design. In this study, eight promising scaffolds were identified for the CXCR2 antagonist with the new pharmacophore we built, among those scaffold **F** was selected for future optimization. In addition, the substituted 1H-1,3,4-triazol derivative **1e** performed well in CXCR2 antagonism and showed good anti-metastatic activity in *in vitro* anti-tumour metastatic assay (figure 2).

## 2. Material and methods

### 2.1. Pharmacophore hypothesis generation and validation

The ‘Common Feature Pharmacophore Generation’ protocol available in Discovery Studio 2.5 (DS 2.5, Accelrys Inc., San Diego, CA, USA) was employed to establish the pharmacophore. The minimum interfeature distance was limited to 1, number of leads that may miss was limited to 0 and the maximum conformation was amplified to 300 by using the ‘best conformers generation’ method with a 20 kcal mol^−1^ energy cut-off, and the default settings were used for the rest of the parameters.

Among the 10 possible hypotheses returned, the top-ranked hypothesis (Hypo 1) was selected and validated by the goodness of hit (GH) scoring method. The decoy set database was made up of 30 active CXCR2 antagonists and other 970 inactive compounds selected from the Maybridge database randomly. The 30 CXCR2 antagonists were different from the compounds in the training set. The ‘Ligand Pharmacophore Mapping’ protocol, with ‘Best’ conformation generation and ‘Flexible’ fitting sets, was used. Hypo 1 was evaluated based on the screening results.

### 2.2. Virtual screening

A screening of the database was performed with the ‘Ligand Pharmacophore Mapping’ protocol implanted in DS 2.5. Maximum omitted features were limited to 0, and the maximum conformations were amplified to 300 by using the ‘best conformers generation’ method with a 20 kcal mol^−1^ energy cut-off, and the default settings were used for the rest of the parameters.

### 2.3. CXC chemokine receptor 2 antagonistic activity assay

Human embryonic kidney 293 (HEK293) cells stably expressing Gα16 and CXCR2 were seeded onto 96-well plates and incubated for 24 h. Cells were loaded with 2 µmol l^−1^ Fluo-4 AM in Hank's balanced salt solution (HBSS, containing KCl 5.4 mmol l^−1^, Na_2_HPO_4_ 0.3 mmol l^−1^, KH_2_PO_4_ 0.4 mmol l^−1^, NaHCO_3_ 4.2 mmol l^−1^, CaCl_2_ 1.3 mmol l^−1^, MgCl_2_ 0.5 mmol l^−1^, Mg_2_SO_4_ 0.6 mmol l^−1^, NaCl 137 mmol l^−1^, BSA 5 g l^−1^, glucose 5.6 mmol l^−1^ and sulfinpyrazone 250 µmol l^−1^, pH 7.4) at 37°C for 45 min. The excess dye was removed and 50 µl of HBSS containing test compounds was added. After incubation at room temperature for 10 min, 25 µl of HBSS containing IL-8 was dispensed into the well using a FlexStation II microplate reader (Molecular Devices, Sunnyvale, CA, USA), and intracellular calcium change was recorded with an excitation wavelength of 485 nm and an emission wavelength of 525 nm. The half maximal inhibitory concentrations (IC_50_) of compounds were determined with the GraphPad Prism software by constructing their dose–response curves.

### 2.4. Anti-proliferation assay

NCI-H1299 cells (provided by Tumor Pharmacology and Endocrine Laboratory, College of Pharmaceutical Sciences, Zhe Jiang University) were seeded in 96-well plates at a density of 4000 cells well^−1^. After 24 h of adherence, cells were incubated with medium alone or medium containing test compounds for 72 h. Four different concentrations (100, 10, 1 and 0.1 µg ml^−1^) of both antagonists were used. Cell proliferation was determined by the thiazolyl blue tetrazolium bromide (MTT) assay. Growth inhibition was calculated as % = [1 − (A/B)] × 100, where A and B were the absorbance of treated and untreated cells, respectively.

### 2.5. Wound healing assay

NCI-H1299 cells (5 × 10^5^ cell well^−1^) were seeded in a six-well tissue culture plate and grown to 90% confluence. After the medium was removed, a gap with constant width was created in the centre of the cell well by scratching the monolayer with a sterile yellow micropipette tip. Cells were then rinsed with phosphate-buffered saline thrice to remove cellular debris and were subsequently exposed to 1‰ dimethyl sulfoxide (DMSO) or 50 µg ml^−1^ of compound **1e**. The wound closure was monitored and photographed at 0, 12, 24, 36 and 48 h with the ImagePro software. The cell migration inhibitory rate was calculated as % = [1 − (0 h wound area − 12, 24, 36 or 48 h wound area)/0 h wound area] × 100.

### 2.6. Chemistry

All reagents and solvents were used as purchased from commercial sources. Chromatography was performed using silica gel (200–300 mesh). All reactions were monitored by thin layer chromatography (TLC), using silica gel plates with fluorescence F254 and ultraviolet light visualization. Proton nuclear magnetic resonance (NMR) spectra were obtained on a Bruker AVII 500 with the use of CDCl_3_, CD_3_OD, (CD_3_)_2_CO or DMSO-d_6_ as solvents. Carbon-13 NMR spectra were obtained on a Bruker spectrometer (125 MHz) by the use of DMSO-d_6_ as a solvent. Chemical shifts are referenced to the residual solvent peak and reported in ppm (*d*-scale) and all coupling constant (*J*) values are given in Hz. The following multiplicity abbreviations are used: (s) singlet, (d) doublet, (t) triplet, (q) quartet, (m) multiplet and (br) broad. Electron spray ionization-mass spectrum (ESI-MS) data were recorded on a Shimadzu LC-MS 2020.

## 3. Results and discussion

### 3.1. Database establishment

**Figure 3.**
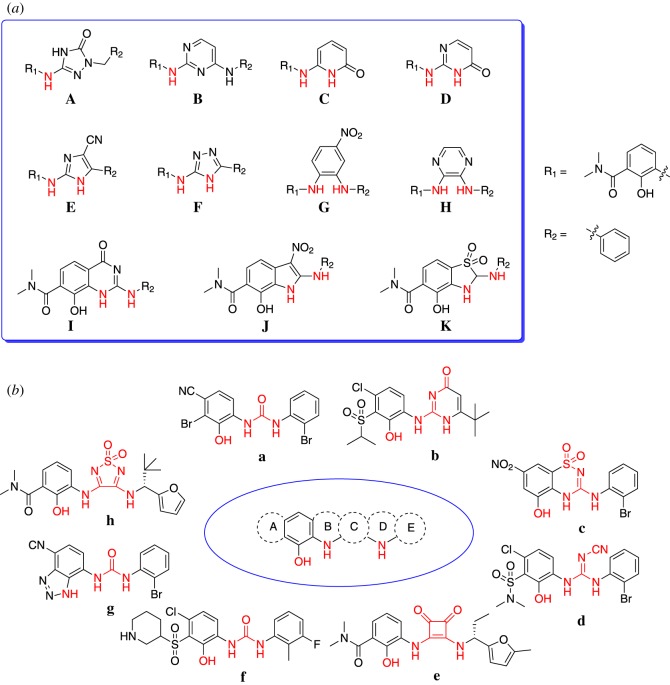
(*a*) Designed compounds in database and (*b*) compounds in the training set used for pharmacophore generation.

To explore novel hit/lead compounds as CXCR2 antagonists, we designed a set of novel scaffolds on the basis of known structure–activity relationships of diarylurea CXCR2 antagonists [14], as shown in figure 3*a*. Scaffolds **A**–**D** were designed based on urea bioisosteres, and scaffolds **E**–**K** contained a five- or six-membered ring with the essential –NHs. It will be inefficient to identify the valid scaffold by synthesizing all the scaffolds and testing their antagonistic activity. Hence, in order to identify valid scaffold efficiently, a pharmacophore model was built as a filtrating tool for virtual screening. For convenience in the screening, the R_1_ group was set to 2-hydroxy-*N*,*N*-dimethylbenzamide-1-yl, and the R_2_ group was set to phenyl.

### 3.2. Establishment and validation of the ligand-based pharmacophore

To select compounds as the training set to establish the pharmacophore model, the following criteria were applied: (i) a certain degree of structure diversity should be shared; (ii) compounds should be the most active from each series; and (iii) compounds should contain similar pharmacophore components in order to ensure the similar binding models against CXCR2 [26]. Eight compounds **a**–**h** fitted with our restrictions and were selected. These compounds shared a prototypical pharmacophore scheme, structurally diverse –NHs and a phenolic hydroxyl group. In addition, the IC_50_ values of the compounds ranged from 1 to 50 nM [27–32] (figure 3*b*).

**Figure 4.**
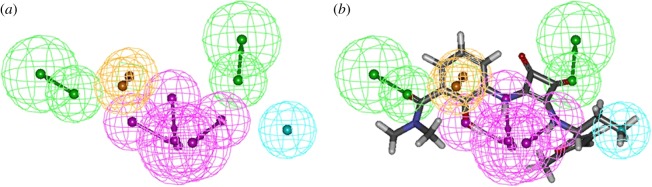
(*a*) Space organization of pharmacophore model and (*b*) highly active antagonist compound **e** mapped to the pharmacophore model as the best mapping result.

The HipHop module implanted in DS 2.5 was adaptively used for establishing the pharmacophore model. The analyses of the chemical features present in the training set structures led to the selection of five features, including hydrogen-bond acceptor (A), hydrogen-bond donor (D), hydrophobe (H), positive ion (PI) and aromatic ring (R). In this study, as the close distance of essential groups, the minimum interfeature distance was limited to 1, number of leads that may miss was limited to 0 and the maximum conformation was amplified to 300 by using the ‘best conformers generation’ method with a 20 kcal mol^−1^ energy cut-off, and the default settings were used for the rest of the parameters, related parameters can be found in the electronic supplementary material, table S1. For the calculation step, the principle and MaxOmitFeat values of all compounds were set as 2 and 1, respectively. Ten pharmacophore models were generated, as shown in table 1, and the best one was selected as Hypo 1 based on the top rank value of 140.981. Hypo 1 consisted of three hydrogen-bond donors (HBDs), two hydrogen-bond acceptors (HBA), a hydrophobic group (HY) and an aromatic ring. As shown in figure 4*b*, the highly active compound **e** (CXCR2 IC_50_ = 2 nM) was perfectly mapped to Hypo 1. Therefore, Hypo 1 was used for further validation.

**Table 1.** The scores of common feature pharmacophore hypotheses (HipHop). (R, aromatic ring; H, hydrophobic; D, H-bond donor; A, H-bond acceptor.)

no.	features	rank	direct hit	fit
1	RHDDDAA	140.981	11111111	7
2	RHDDDAA	140.700	11111111	7
3	RHDDDAA	140.700	11111111	7
4	RHDDDAA	139.098	11111111	7
5	RHDDDAA	136.997	11111111	7
6	RHDDDAA	136.616	11111111	7
7	RHDDAAA	135.016	11111111	7
8	RHDDAAA	135.016	11111111	7
9	RHDDDAA	134.033	11111111	7
10	RHDDDAA	131.672	11111111	7

Hypo 1 was further validated by the GH scoring method [33]. A decoy set database used for pharmacophore validation was made up of 30 independent active compounds and 970 inactive compounds which were chosen randomly from the Maybridge database. Among 30 hits, 24 positive compounds were successfully identified. A set of statistical parameters, such as yield of actives, ratio of actives, enrichment factor (EF) and GH scores [26], are presented in table 2. As a consequence, Hypo 1 was a good candidate for conducting virtual screening.

**Table 2.** Statistical parameters and scores of the study for validation of Hypo 1.

no.	parameters	values
1	total molecules in database (D)	1000
2	total number of actives in database (A)	30
3	total hits (Ht)	30
4	active hits (Ha)	24
5	% yield of actives [(Ha/Ht) × 100]	80.0%
6	% ratio of actives [(Ha/A) × 100]	80.0%
7	enrichment factor (E) [(Ha × D)/(Ht × A)]	26.7
8	false negatives [A − Ha]	6
9	false positive [Ht − Ha]	6
10	goodness of hit (GH) ^a^^,^^b^	0.79

^a^[Ha/(4 × Ht × A)] × (3 × A + Ht) × [1 − (Ht − Ha)/(D − A)].

^b^GH score 0.6–0.8 indicates a very good model.

### 3.3. Virtual screening

**Figure 5.**
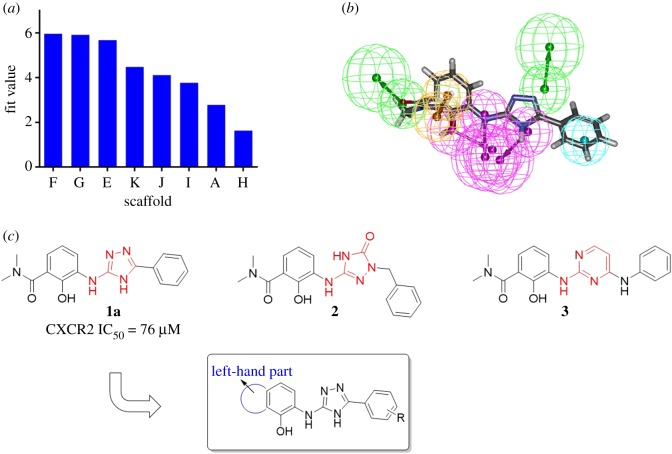
(*a*) Fit value of different scaffolds; (*b*) compound **1a** bearing scaffold **F** mapped to Hypo 1; and (*c*) structures of **1a**, **2** and **3**.

After screening the database with the Hypo 1 (parameters can be found in the electronic supplementary material, table S2), eight hits were identified, as shown in figure 5*a*. Scaffolds **B**–**D** showed no mapping results, scaffold **F** exhibited the best-fit value of 5.95807, whereas scaffolds **G** and **E** performed less well. Compound **1a** bearing scaffold **F** was mapped well with all pharmacophore elements (figure 5*b*). The hydroxyl phenolic group and the two NHs functioned as hydrogen-bond donors, and the 1-N on 1,2,4-triazole core functioned as a hydrogen-bond acceptor. Thus, scaffold **F** was chosen for further exploration.

As expected, compound **1a** showed a CXCR2 IC_50_ value of 76 µM, which was believed to be a novel privileged scaffold for CXCR2 antagonism and selected for further optimization and biological evaluation. In addition, compound **2** bearing scaffold **A** exhibited low-fit value and compound **3** bearing scaffold **B** exhibited no mapping result, and both were also chosen to be synthesized as control compounds. Moreover, former reported articles [27–32] and our group have noted that introducing an electron-withdrawing group to the left-hand part was favouring compounds’ antagonistic activity; therefore, the following optimization of scaffold **F** was mainly focused on the left-hand amide group to identify more potent antagonists (figure 5*c*). A cyano group and sulfonyl group were introduced to the left-hand part as privileged electron-withdrawing groups.

### 3.4. Chemistry

**Figure 6.**
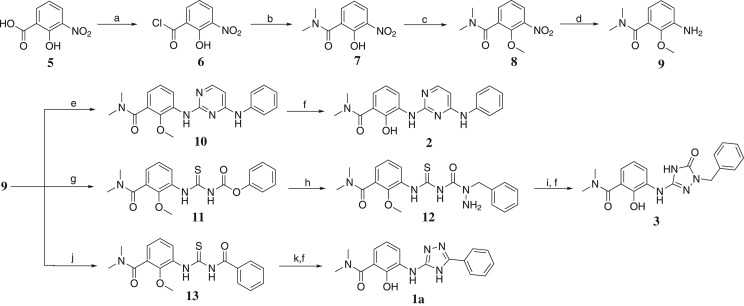
Modification of the right-hand part. Reagents and conditions: a, thionyl chloride, reflux; b, dimethylamine hydrochloride, DIPEA, CH_2_Cl_2_; c, dimethyl sulfate, K_2_CO_3_, acetone; d, Fe, NH_4_Cl, EtOH/H_2_O; e, 2-chloro-*N*-phenylpyrimidin-4-amine, Pd(OAc)_2_, Cs_2_CO_3_, Xanphos, 1,4-dioxane; f, 1 M BBr_3_, CH_2_Cl_2_; g, KSCN, phenyl chloroformate, acetone; h, benzylhydrazine dihydrochloride, MeOH; i, EtOH, reflux; j, KSCN, benzoyl chloride, acetone; and k, hydrazine hydrate, EtOH.

The synthesis of compounds **1a**, **2** and **3** is shown in figure 6. The substituted aniline (compound **9**) used as an important intermediate was derived from commercial available starting material compound **5**, with the following steps: acylation (**6**), condensation (**7**), methyl substitution (**8**) and reduction. Compound **9** underwent a palladium-catalysed coupling reaction with 2-chloro-*N*-phenylpyrimidin-4-amine to provide the substituted pyrimidine compound **10**. Then, deprotection of compound **10** in the presence of 1 M BBr_3_ furnished target compound **2**. Thiourea compounds **11** and **13** were prepared by using potassium thiocyanate and phenyl chloroformate or benzoyl chloride, respectively. In addition, compound **12** was obtained by the nucleophilic substitution of **11** with benzylhydrazine and subsequently used to synthesize compound **5** via a cyclic reaction and deprotection. In addition, the preparation of the triazol compound was started with a cyclic reaction of **13** by the presence of hydrazine hydrate in ethanol at reflux condition, and then the deprotection of a methyl group gave compound **1a**.

**Figure 7.**
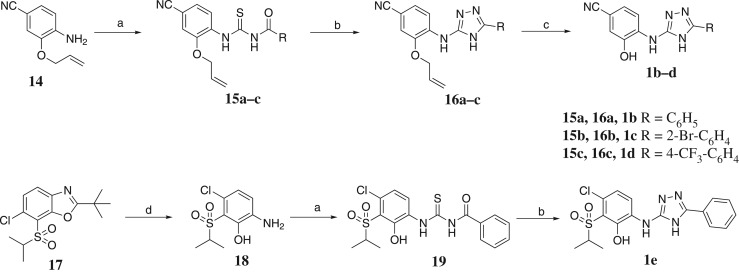
Modification of the left-hand part. Reagents and conditions: a, KSCN, appropriate acyl chloride; b, hydrazine hydrate, EtOH; c, Pd(PPh_3_)_4_, morpholine; and d, concentrated HCl, EtOH, reflux.

The synthesis of 4H-1,2,4-triazol derivatives **1b–e** is illustrated in figure 7. Having noted that the 4H-1,2,4-triazol scaffold was not stable under highly acidic conditions when deprotection of the methyl group was occurring (after changing the left-hand amide group to cyano group), the methyl protection was replaced with allyl protection, which can be removed under milder conditions. Compound **14** was used as the starting material, and compounds **16a–c** were obtained by the above method of establishing 4H-1,2,4-triazol scaffold. The deprotection of **16a–c** in the presence of the Pd(PPh_3_)_4_/piperidine system resulted in target compounds **1b–d**. In addition, compound **17** was ring-opened under a concentrated HCl condition to the corresponding sulfonyl containing aromatic amine compound **18**, and compound **18** was subsequently reacted with potassium thiocyanate and benzoyl chloride to give thiourea compound **19**, and then direct cyclized with hydrazine hydrate to afford the final product **1e**.

### 3.5. *In vitro* CXC chemokine receptor 2 antagonistic activity evaluation

The CXCR2 antagonist **MK7123** was used as the standard drug during the *in vitro* activity test. Compounds **2** and **3** bearing urea bioisoteres scaffold exhibited the loss of CXCR2 antagonistic activity. Furthermore, we thought that introducing a stronger electron-withdrawing group to improve the acidity (p*K*_a_) of –NHs on the triazole moiety may potentially provide stronger H-bonding interactions with CXCR2 [32]. At first, the cyano group was introduced to the R_2_ position (**1b**), which resulted in an improvement of the CXCR2 IC_50_ value of 18.5 µM as expected. However, introducing neither halogen (**1c**) nor trifluoromethyl (**1d**) to the right-hand side phenyl enhanced its CXCR2 antagonistic potency. Sulfonyl, which is an another suitable group applied in other CXCR2 antagonists, also showed a positive effect when applied in 4H-1,2,4-triazole scaffold, the IC_50_ value of compound **1e**, in fact, reached 14.8 µM, as shown in table 3. As expected, introducing stronger electron-withdrawing groups to the left phenol ring can improve the CXCR2-binding affinity of compounds.

**Table 3.** Antagonistic activity of synthesized compounds against CXCR2.

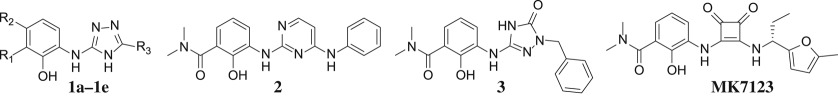						
no.	R_1_	R_2_	R_3_	CXCR2 IC_50_ (μM)	s.e.m.	fit value
**1a**	–CON(CH_3_)_2_	H	–C_6_H_5_	76	5.6	5.95807
**2**	—	—	—	>100	—	2.78005
**3**	—	—	—	>100	—	—
**1b**	H	CN	–C_6_H_5_	18.5	3.7	4.69647
**1c**	H	CN	2-Br-C_6_H_4_	25.4	8.6	4.81771
**1d**	H	CN	4-CF_3_-C_6_H_4_	>100	—	4.87115
**1e**	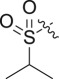	Cl	–C_6_H_5_	14.8	9.6	5.98048
**MK7123**	—	—	—	0.008	0.003	6.99950

The fit value represents how well a compound maps to the pharmacophore, the higher the fit value, the better the performance on CXCR2 antagonism would be. For example, comparing compound **2** or **3**, compound **1e** exhibited a better-fit value of 5.98048 accompanied with better CXCR2 antagonistic potency. In addition, compound **2** had the worst performance, which indicated a loss of CXCR2 IC_50_. However, when a minor change occurred on the scaffold, like in the case of compound **1b–1d**, other factors, such as the electron cloud distribution of the whole molecule and the space of protein-binding pocket, could present more impact on the compounds' antagonistic potential against CXCR2. Under this circumstance, the compound IC_50_ may not follow the fit value, and other computational data support will be necessary.

### 3.6. Compound **1e** suppressed cell survival and migration

**Figure 8.**
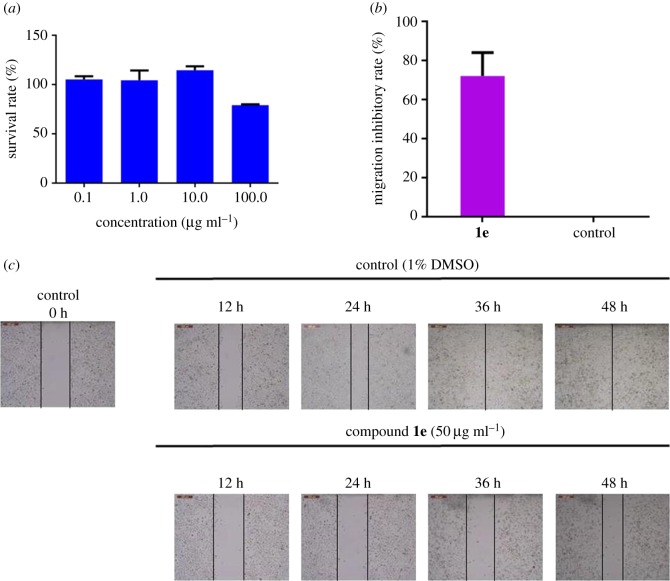
(*a*) The cytotoxicity of compound **1e**. (*b*) The migration inhibitory rate of compound **1e** and the control group. (*c*) The images of NCI-H1299 cell migration.

The highly metastatic NCI-H1299 lung cancer cell line was selected for the following *in vitro* biological evaluation. The migration inhibitory effect of compound **1e** was studied based on a well-established wound healing assay. At first, the cytotoxicity of compound **1e** was evaluated to provide a suitable concentration range for the cell migration assays. The cell line was treated with increasing concentrations of compound **1e** (0.1, 1, 10 and 100 µg ml^−1^) for 72 h, then growth inhibition was measured by the MTT assay. As shown in figure 8*a*, compound **1e** displayed no cytotoxicity at various concentrations, which will not induce cell death during the migration assay.

Based on the cytotoxicity results, cells were treated with 1‰ DMSO or 50 µg ml^−1^ of compound **1e** for 48 h and photographed every 12 h. The wound area was calculated with the ImagePro software. The result indicated that the wound area showed a time-dependent decrease in healing, and the dosing group exhibited a higher migration inhibitory rate (compound **1e** 72 ± 12%) than the control group after 48 h treatment, as shown in figure 8*b*,*c*. Therefore, it is believed that compound **1e** may modulate the migration capacity of NCI-H1299 cell line *in vitro*. Targeting CXCR2 may provide a more effective approach for anti-cancer metastasis treatment.

## 4. Conclusion

In summary, a reliable ligand-based pharmacophore model Hypo 1 was successfully constructed and validated to identify novel potent CXCR2 antagonists from the database based on the known SAR of diarylurea analogues. Eight hit compounds were identified, compound **1a** was selected for the best performance. A 4H-1,2,4-triazol scaffold was chosen, synthesized and subjected to the *in vitro* biological test which led to a positive outcome. Further structural optimization led to the discovery of compound **1e**. Compound **1e** was further studied for its high anti-metastatic potency in the NCI-H1299 cell line, suggesting that it could be used as a good hit compound for CXCR2 antagonism and as an anti-cancer metastatic agent. The pharmacophore model Hypo 1 will be applied to screen other larger databases, the target validation and optimization of compound **1e** is in progress and will be reported in further works.

## Supplementary Material

Parameters of pharmacophore hypothesis generation; Parameters of validation and screening; Chemistry; NMR Spectrum of compounds

## Data accessibility

This article does not contain any additional data.
