# Supplementary material for: Ligand-based pharmacophore model for the discovery of novel CXCR2 antagonists as anti-cancer metastatic agents
Source: R Soc Open Sci. 2018 Jul 4;5(7):180176. doi: 10.1098/rsos.180176 (PMC6083693; doi:10.1098/rsos.180176)

**Electronic supplementary material**

**Ligand-based Pharmacophore Model for the discovery of Novel CXCR2 Antagonists as Anti-cancer Metastatic Agents**

Jin-Xin Che^1, ‡^, Zhi-Long Wang^2, ‡^, Hai-Chao Sheng^1^, Feng Huang^1^, Xiao-Wu Dong^1^, You-Hong Hu^2^, Xin Xie^2, 3, *^ and Yong-Zhou Hu^1, *^

^1^ *ZJU-ENS Joint Laboratory of Medicinal Chemistry, College of Pharmaceutical Sciences, Zhejiang University, Hangzhou, PR China*

^2^ *State Key Laborarory of Drug Research, Shanghai Institute of Materia Medica, Chinese Academy of Sciences, Shanghai, PR China*

^3^ *CAS Key Laboratory of Receptor Research, the National Center for Drug Screening, Shanghai Institute of Materia Medica, Chinese Academy of Sciences, Shanghai, PR China*

^‡^ These authors contributed equally.

^*^ Corresponding authors.

Email addresses: [huyz@zju.edu.cn](mailto:huyz@zju.edu.cn) (Yong-Zhou Hu);

[xxie@mail.shcnc.ac.cn](mailto:xxie@mail.shcnc.ac.cn) (Xin Xie)

**Table of Contents**

[Table S1. Parameters of pharmacophore hypothesis generation 2](#_Toc505328146)

[Table S2. Parameters of validation and screening 3](#_Toc505328147)

[Chemistry 4](#_Toc505328148)

[NMR Spectrum of compounds 8](#_Toc505328149)

# Table S1. Parameters of pharmacophore hypothesis generation

| **Items** | **Parameters** |
| --- | --- |
| Features | HB_ACCEPTOR 0 5, HB_DONOR 0 5, HYDROPHOBIC 0 5, POS_IONIZABLE 0 5, RING_AROMATIC 0 5 |
| Maximum Pharmacophores | 10 |
| Minimum Features | 1 |
| Maximum Features | 10 |
| Minimum Interfeature Distance | 1 |
| Number of Leads That May Miss | 0 |
| Maximum Excluded Volumes | 0 |
| Minimum Feature Points | 4 |
| Minimum Features in Moderately Active | 4 |
| Conformation Generation | BEST |
| Maximum Conformations | 300 |
| Discard Existing Conformations | False |
| Energy Threshold | 20.0 |
| Ring Fragments File |  |
| Save Conformations | False |
| Parallel Processing | False |
| Parallel Processing Batch Size | 25 |
| Parallel Processing Server | localhost |
| Parallel Processing Server Processes | 2 |
| Parallel Processing Preserve Order | True |
| Spreadsheet File |  |
| Feature Misses | 1 |
| Complete Misses | 0 |
| Align Ligands to Hypothesis | True |
| Best Mapping Only | True |
| Check Superposition | True |
| Superposition Error | 1.0 |
| Scale Feature Blob Size | 1.0 |
| Maximum Mappings | 1000 |
| Maximum Confs Per Mapping | 1000 |
| Memory | 240 |
| Explore Exhaustive HBond Geometry | True |
| Catalyst Parameter File |  |
| CustomDictionary |  |

# Table S2. Parameters of validation and screening

| **Items** | **Parameters** |
| --- | --- |
| Input Database Limit Hits | All |
| Input Database Maximum | 300 |
| Input Database Hitlist |  |
| NOT Features |  |
| Best Mapping Only | True |
| Maximum Omitted Features | 0 |
| Fitting Method | Flexible |
| Conformation Generation | BEST |
| Maximum Conformations | 300 |
| Discard Existing Conformations | True |
| Energy Threshold | 20.0 |
| Ring Fragments File |  |
| Save Conformations | True |
| Property Names |  |
| Fit Name | FitValue |
| Shape Name | ShapeSimilarity |
| Estimate Name | Estimate |
| MapID Name | MappingID |
| Pharmprint Name | Pharmprint |
| Pharmtype Name | Pharmtype |
| Minimum Interfeature Distance | 2.0 |
| Map Each Conformation Separately | False |
| Specify Energy Threshold | False |
| Mapping Energy Threshold | 20.0 |
| Output Non-Fitting Ligands | False |
| Scale Fit Values | False |
| Sort Fit Values | True |
| Limit Hits | All |
| Maximum Hits | 300 |
| Keep Per Molecule | 999 |
| Catalyst Parameter File |  |
| Parallel Processing | False |
| Parallel Processing Batch Size | 25 |
| Parallel Processing Server | localhost |
| Parallel Processing Server Processes | 2 |
| Parallel Processing Preserve Order | True |
| FilesToOpen | ViewResults.pl |

# Chemistry

**Preparation of 3-amino-2-methoxy-N,N-dimethylbenzamide (9)**

2-hydroxy-3-nitrobenzoic acid (compound 5, 5 g) was added to thionyl chloride (5 mL) and heated to reflux for 12h. The solution was concentrated to afford 2-hydroxy-3-nitrobenzoyl chloride (compound 6, 4.6 g) which can be directly used for the next step. To a solution of 6 (4 g, 20 mmol) and TEA (8 g, 80 mmol) in DCM was added dimethylamine hydrochloride (3.3 g, 40 mmol), then stirred for 6h at room temperature. The solution was extracted with 1N NaOH (20 mL × 3), the aqueous phase was combined and acidized to PH = 1~2 with 1N HCl, then extracted with EtOAc (50 mL × 3). The combined organic layers were washed with brine, dried over Na_2_SO_4_, filtered and concentrated to afford compound **7** (3.6 g).

To a solution of **7** (3.15 g, 15 mmol) in acetone was added K_2_CO_3_ (3.1 g, 22.5 mmol), then stirred for 0.5h at 0℃. Then dimethyl sulfate (1.9 g, 15 mmol) was added slowly, and the mixture was stirred at room temperature for 5h, then filtered and concentrated. The residue was diluted with EtOAc (100 mL), washed with 1N NaOH (20 mL × 3), brine and dried over Na_2_SO_4_, filtered and concentrated to afford compound **8** (2.8 g).

To a solution of **8** (3.36 g, 15 mmol), NH_4_Cl (3.24 g, 60 mmol) in EtOH/H_2_O = 3:1 was added Fe powder (6.72 g, 120 mmol), then stirred for 12h at reflux condition. The solution was concentrated, the residue was dissolved in water, then basified with 1N NaOH to PH = 12~13. The mixture was extracted with EtOAc (50 mL × 3). The combined organic layers were washed with brine, dried over Na_2_SO_4_, filtered and concentrated. The residue was purified by silica gel chromatography to afford compound **9** (1.2 g, 25% total yield) as light yellow liquid.^1^H NMR (500 MHz, CDCl_3_) δ 6.95 – 6.90 (m, 1H), 6.76 (m, 1H), 6.62 (m, 1H), 3.79 (s, 3H), 3.13 (s, 3H), 2.88 (s, 3H). ESI-MS: *m/z* =225 [M+H] ^+^.

**Preparation of 2-methoxy-N,N-dimethyl-3-((4-(phenylamino)pyrimidin-2-yl)amino)benzamide (10)**

To a solution of **9** (97 mg, 0.5 mmol), 2-chloro-N-phenylpyrimidin-4-amine (144 mg, 0.7 mmol), Pd(OAc)_2_ (15 mg, 0.065 mmol), Cs_2_CO_3_ (326 mg, 1 mmol) and 4,5-Bis(diphenylphosphino)-9,9-dimethylxanthene (72 mg, 0.125 mmol) in anhydrous 1,4-dioxane, then heated to 160℃ with microwave under N_2_ protection for 20 min. The solution was quenched with water, and then the mixture was extracted with EtOAc (30 mL × 3). The combined organic layers were washed with brine, dried over Na_2_SO_4_, filtered and concentrated. The residue was purified by silica gel chromatography to afford compound **10** (100 mg, yield 55%). ^1^H NMR (500 MHz, CDCl_3_) δ 8.46 (d, *J* = 8.0 Hz, 1H), 8.01 (d, *J* = 5.5 Hz, 1H), 7.39 (m, 4H), 7.22 – 7.17 (m, 1H), 7.10 (t, *J* = 8.0 Hz, 1H), 6.89 (m, 1H), 6.24 (d, *J* = 6.0 Hz, 1H), 3.86 (s, 3H), 3.15 (s, 3H), 2.89 (s, 3H). ESI-MS: *m/z* =364 [M+H] ^+^.

**Preparation of 2-methoxy-N,N-dimethyl-3-(3-(phenoxycarbonyl)thioureido)benzamide (11)**

To a solution of phenyl chloroformate (513 mg, 3 mmol) in acetone was added KSCN (310 mg, 3.2 mmol) slowly. The mixture was stirred at room temperature for 0.5h, and then compound **9** (194 mg, 1 mmol) was added. After compound **9** was consumed, the solution was concentrated and the residue was diluted with 0.5N HCl. The mixture was extracted with EtOAc (50 mL × 3). The combined organic layers were washed with brine, dried over Na_2_SO_4_, filtered and concentrated. The residue was purified by silica gel chromatography to afford compound **11** (330 mg, yield 88%). ^1^H NMR (500 MHz, CDCl_3_) δ 7.43 (m, 2H), 7.31 (m, 1H), 7.23 – 7.11 (m, 5H), 3.82 (s, 3H), 3.13 (s, 3H), 2.86 (s, 3H). ESI-MS: *m/z* =374 [M+H] ^+^.

**Preparation of 1-benzyl-N-((3-(dimethylcarbamoyl)-2-methoxyphenyl)carbamothioyl) hydrazine-1-carboxamide (12)**

To a solution of compound **11** (186 mg, 0.5 mmol), DIPEA (387 mg, 3 mmol) in THF was added benzylhydrazine dihydrochloride (293 mg, 1.5 mmol), the mixture was stirred at room temperature for 12h. The solution was concentrated, and the residue was diluted with EtOAc (100 mL), washed with 1N HCl (20 mL × 3), brine and dried over Na_2_SO_4_, filtered and concentrated. The residue was purified by silica gel chromatography to afford compound **12** (127 mg, yield 63%). ^1^H NMR (500 MHz, CDCl_3_) δ 12.78 (s, 1H), 9.90 (s, 1H), 8.84 (m, 1H), 7.43 – 7.36 (m, 3H), 7.31 – 7.28 (m, 2H), 7.19 (m, 1H), 7.11 (m, 1H), 4.76 (s, 2H), 3.89 (s, 3H), 3.14 (s, 3H), 2.89 (s, 3H). ESI-MS: *m/z* =402 [M+H] ^+^.

**Preparation of 3-(3-benzoylthioureido)-2-methoxy-N,N-dimethylbenzamide (13)**

To a solution of benzoyl chloride (210 mg, 1.5 mmol) in acetone was added KSCN (155 mg, 1.6 mmol) slowly. The mixture was stirred at room temperature for 0.5h, and then compound **9** (97 mg, 0.5 mmol) was added. After compound **9** was consumed, the solution was concentrated and the residue was diluted with water. The mixture was extracted with EtOAc (50 mL × 3). The combined organic layers were washed with brine, dried over Na_2_SO_4_, filtered and concentrated. The residue was purified by silica gel chromatography to afford compound **13** (136 mg, yield 76%). ^1^H NMR (500 MHz, CDCl_3_) δ 13.11 (s, 1H), 9.08 (s, 1H), 8.91 (m, 1H), 7.95 – 7.89 (m, 2H), 7.66 (m, 1H), 7.56 (m, 2H), 7.22 (t, *J* = 8.0 Hz, 1H), 7.16 (m, 1H), 3.93 (s, 3H), 3.16 (s, 3H), 2.90 (s, 3H). ESI-MS: *m/z* =358 [M+H] ^+^.

**General procedure A for preparation of 3-(allyloxy)-4-((5-substituted-4H-1,2,4-triazol-3-yl)amino)benzonitrile (16a-c)**

To a solution of substituted benzoyl chloride (1.5 mmol) in acetone was added KSCN (1.6 mmol) slowly. The mixture was stirred at room temperature for 0.5h, and then compound **14** (0.5 mmol) was added. The mixture was continuing stirred at room temperature for 12h, lots of solid precipitated out, then the precipitation was filtered, washed with cool acetone and dried in oven to afford compound **15a-c** which can be directly used to the next step.

To a solution of **15a-c** (0.5 mmol) in ethanol was added hydrazine hydrate (0.75 mmol), the mixture was stirred to reflux for 12h. The solution was concentrated and the residue was diluted with water. The mixture was extracted with EtOAc (50 mL × 3). The combined organic layers were washed with brine, dried over Na_2_SO_4_, filtered and concentrated. The residue was purified by silica gel chromatography to afford compound **16a-c**.

**3-(allyloxy)-4-((5-phenyl-4H-1,2,4-triazol-3-yl)amino)benzonitrile (16a).**

General procedure A, yield: 39%; ^1^H NMR (500 MHz, Acetone) δ 13.16 (br, 1H), 8.55 (br, 1H), 8.09 (m, 2H), 7.52 (m, 3H), 7.45 – 7.30 (m, 3H), 6.28 – 6.10 (m, 1H), 5.52 (m, 1H), 5.36 (d, *J* = 10.5 Hz, 1H), 4.84 (d, *J* = 5.5 Hz, 2H). ESI-MS: *m/z* =318 [M+H] ^+^.

**3-(allyloxy)-4-((5-(2-bromophenyl)-4H-1,2,4-triazol-3-yl)amino)benzonitrile (16b).**

General procedure A, yield: 32%; ^1^H NMR (500 MHz, DMSO) δ 7.86 – 7.47 (m, 5H), 7.43 (m, 2H), 6.13 (s, 1H), 5.47 (d, *J* = 17.0 Hz, 1H), 5.33 (d, *J* = 9.5 Hz, 1H), 4.79 (s, 2H). ESI-MS: *m/z* =396 [M+H] ^+^.

**3-(allyloxy)-4-((5-(4-(trifluoromethyl)phenyl)-4H-1,2,4-triazol-3-yl)amino)benzonitrile (16c).**

General procedure A, yield: 42%; ^1^H NMR (500 MHz, DMSO) δ 8.22 (m, 2H), 7.87 (m, 3H), 7.46 (m, 2H), 6.13 (m, 1H), 5.48 (m, 1H), 5.34 (d, *J* = 10.5 Hz, 1H), 4.79 (s, 2H). ESI-MS: *m/z* =386 [M+H] ^+^.

**Preparation of 6-amino-3-chloro-2-(isopropylsulfonyl)phenol (18)**

To a solution of compound **17** (315 mg, 1 mmol) in ethanol (30mL) was added concentrated HCl (30 mL), and heated to reflux for 12h. The solution was concentrated and diluted with water, then basified with 1N NaOH to PH = 7~8. The mixture was extracted with EtOAc (50 mL × 3). The combined organic layers were washed with brine, dried over Na_2_SO_4_, filtered and concentrated. The residue was purified by silica gel chromatography to afford compound **18** (227 mg, yield 91%). ^1^H NMR (500 MHz, CDCl_3_) δ 10.61 (s, 1H), 6.86 (d, *J* = 8.5 Hz, 1H), 6.81 (d, *J* = 8.5 Hz, 1H), 3.87 (m, 1H), 1.38 (d, *J* = 7.0 Hz, 6H). ESI-MS: *m/z* =250 [M+H] ^+^.

**Preparation of N-((4-chloro-2-hydroxy-3-(isopropylsulfonyl) phenyl) carbamothioyl)benzamide (19)**

To a solution of benzoyl chloride (140 mg, 1 mmol) in acetone was added KSCN (97 mg, 1 mmol) slowly. The mixture was stirred at room temperature for 0.5h, and then compound **18** (249 mg, 1 mmol) was added. After compound **18** was consumed, the solution was concentrated and the residue was diluted with water. The mixture was extracted with EtOAc (50 mL × 3). The combined organic layers were washed with brine, dried over Na_2_SO_4_, filtered and concentrated. The residue was purified by silica gel chromatography to afford compound **19** (331 mg, yield 80%). ^1^H NMR (500 MHz, CDCl_3_) δ 13.14 (s, 1H), 11.22 (s, 1H), 9.20 (d, *J* = 9.0 Hz, 1H), 9.09 (s, 1H), 7.95 – 7.90 (m, 2H), 7.71 – 7.64 (m, 1H), 7.56 (m, 2H), 7.12 (d, *J* = 9.0 Hz, 1H), 3.89 (m, 1H), 1.41 (d, *J* = 7.0 Hz, 6H). ESI-MS: *m/z* =413 [M+H] ^+^.

**General procedure B for deprotection of methyl (2, 3, 1a)**

To a solution of methyl protected compounds (0.2 mmol) in anhydrous dichloromethane was added 1M BBr_3_ (0.6 mmol) under N_2_ protection at 0℃, then the mixture was stirred at room temperature for 3h. The solution was basified with saturated NaHCO3 and concentrated. The residue was diluted with water, and extracted with EtOAc (50 mL × 3). The combined organic layers were washed with brine, dried over Na_2_SO_4_, filtered and concentrated. The residue was purified by silica gel chromatography to afford compound **2**, **3** or **1a**.

**2-hydroxy-N,N-dimethyl-3-((4-(phenylamino)pyrimidin-2-yl)amino)benzamide (2).**

General procedure B, yield: 46%; ^1^H NMR (500 MHz, CDCl_3_) δ 7.78 (d, J = 6.0 Hz, 1H), 7.37 (d, J = 8.0 Hz, 1H), 7.30 (m, 2H), 7.26 (m, 2H), 7.04 (t, J = 7.0 Hz, 1H), 6.86 (m, 1H), 6.76 (t, J = 8.0 Hz, 1H), 6.08 (d, J = 6.0 Hz, 1H), 2.97 (s, 6H). ESI-MS: *m/z* =348 [M-H] ^+^.

**3-((1-benzyl-5-oxo-4,5-dihydro-1H-1,2,4-triazol-3-yl)amino)-2-hydroxy-N,N-dimethylbenzamide (3).**

To a solution of compound **12** (80 mg, 0.2 mmol) in ethanol was heated to reflux for 12h. The solution was concentrated and diluted with water. The mixture was extracted with EtOAc (50 mL × 3). The combined organic layers were washed with brine, dried over Na_2_SO_4_, filtered and concentrated. The products can be directly used in the next step without further purification. Then follow the General procedure B for deprotection of methyl. Yield: 32%; ^1^H NMR (500 MHz, MeOD) δ 7.82 (m, 1H), 7.32 (m, 4H), 7.26 (m, 1H), 6.89 – 6.85 (m, 2H), 4.86 (s, 2H), 3.06 (s, 6H). ^13^C NMR (126 MHz, MeOD) δ 177.95, 169.45, 152.24, 149.93, 148.06, 131.77, 129.61, 129.30, 126.16, 124.80, 124.67, 123.61, 121.29, 61.33, 37.86, 34.11. ESI-MS: *m/z* =352 [M-H] ^+^.

**2-hydroxy-N,N-dimethyl-3-((5-phenyl-4H-1,2,4-triazol-3-yl)amino)benzamide (1a).**

General procedure B, yield: 26%; ^1^H NMR (500 MHz, CDCl_3_) δ 8.05 – 7.90 (m, 3H), 7.54 – 7.43 (m, 3H), 6.98 – 6.87 (m, 2H), 3.15 (s, 6H). ESI-MS: *m/z* =322 [M-H] ^+^.

**General procedure C for deprotection of allyl (1b-d)**

To a solution of allyl protected compounds (0.5 mmol) in dichloromethane was added morpholine (0.5 mmol) and Pd(PPh_3_)_4_ (0.04 mmol) under N2 protection. The mixture was stirred at room temperature for 3h, and then concentrated. The residue was diluted with EtOAc (60 mL), washed with 1N HCl (20 mL × 3), brine and dried over Na_2_SO_4_, filtered and concentrated. The residue was purified by silica gel chromatography to afford compound **1b-d**.

**3-hydroxy-4-((5-phenyl-4H-1,2,4-triazol-3-yl)amino)benzonitrile (1b).**

General procedure C, yield: 35%; ^1^H NMR (500 MHz, MeOD) δ 8.32 (d, J = 7.0 Hz, 1H), 7.99 (s, 2H), 7.52 (m, 3H), 7.26 (m, 1H), 7.08 (d, J = 1.5 Hz, 1H). ^13^C NMR (126 MHz, DMSO) δ 129.38, 126.27, 125.37, 120.23, 116.50. ESI-MS: *m/z* =376 [M-H] ^+^.

**4-((5-(2-bromophenyl)-4H-1,2,4-triazol-3-yl)amino)-3-hydroxybenzonitrile (1c).**

General procedure C, yield: 32%; ^1^H NMR (500 MHz, MeOD) δ 8.29 (d, J = 8.5 Hz, 1H), 7.80 (d, J = 8.0 Hz, 1H), 7.73 (d, J = 6.5 Hz, 1H), 7.53 (t, J = 7.5 Hz, 1H), 7.45 (t, J = 7.5 Hz, 1H), 7.24 (m, 1H), 7.08 (d, J = 1.5 Hz, 1H). ESI-MS: *m/z* =354 [M-H] ^+^.

**3-hydroxy-4-((5-(4-(trifluoromethyl)phenyl)-4H-1,2,4-triazol-3-yl)amino)benzonitrile (1d).**

General procedure C, yield: 36%; ^1^H NMR (500 MHz, MeOD) δ 8.38 (d, J = 6.0 Hz, 1H), 8.21 (d, J = 7.5 Hz, 2H), 7.81 (d, J = 8.0 Hz, 2H), 7.26 (m, 1H), 7.08 (d, J = 1.5 Hz, 1H). ^13^C NMR (126 MHz, MeOD) δ 126.38, 125.45, 124.72, 119.23, 115.72. ESI-MS: *m/z* =344 [M-H] ^+^.

**Preparation of 3-chloro-2-(isopropylsulfonyl)-6-((5-phenyl-4H-1,2,4-triazol-3-yl)amino)phenol (1e)**

To a solution of compound **19** (206 mg, 0.5 mmol) in ethanol was added hydrazine hydrate (1.25 mmol), the solution was heated to reflux for 12h. The mixture was concentrated and diluted with water, then acidized with 1N HCl to PH = 5~6. The mixture was extracted with EtOAc (50 mL × 3). The combined organic layers were washed with brine, dried over Na_2_SO_4_, filtered and concentrated. The residue was purified by silica gel chromatography to afford compound **1e** (96 mg, yield 49%). ^1^H NMR (500 MHz, Acetone) δ 11.06 (s, 1H), 8.73 (br, 1H), 8.09 (d, J = 7.5 Hz, 2H), 7.57 – 7.41 (m, 3H), 7.22 (m, 1H), 3.97 (m, 1H), 1.38 (d, J = 7.0 Hz, 6H). ^13^C NMR (125 MHz, DMSO) δ 148.37, 129.85, 126.80, 123.91, 116.97, 55.97, 15.18. ESI-MS: *m/z* =391 [M-H] ^+^.

# NMR Spectrum of compounds


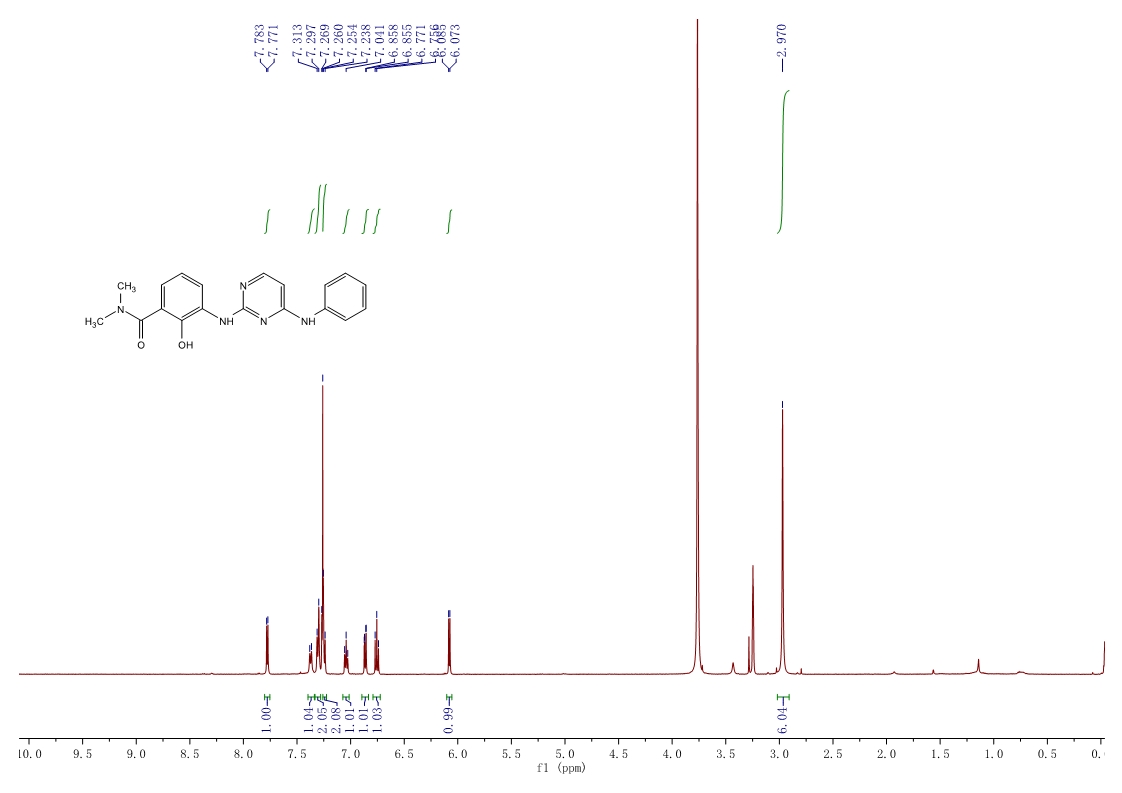


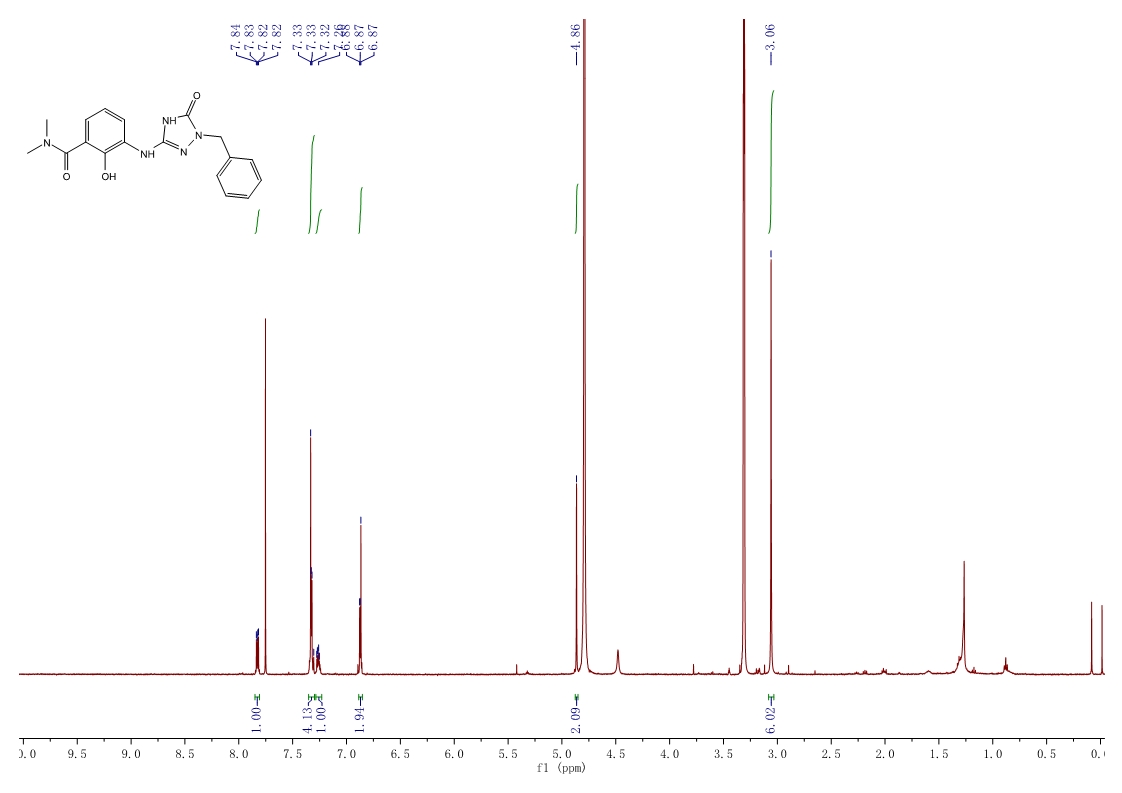


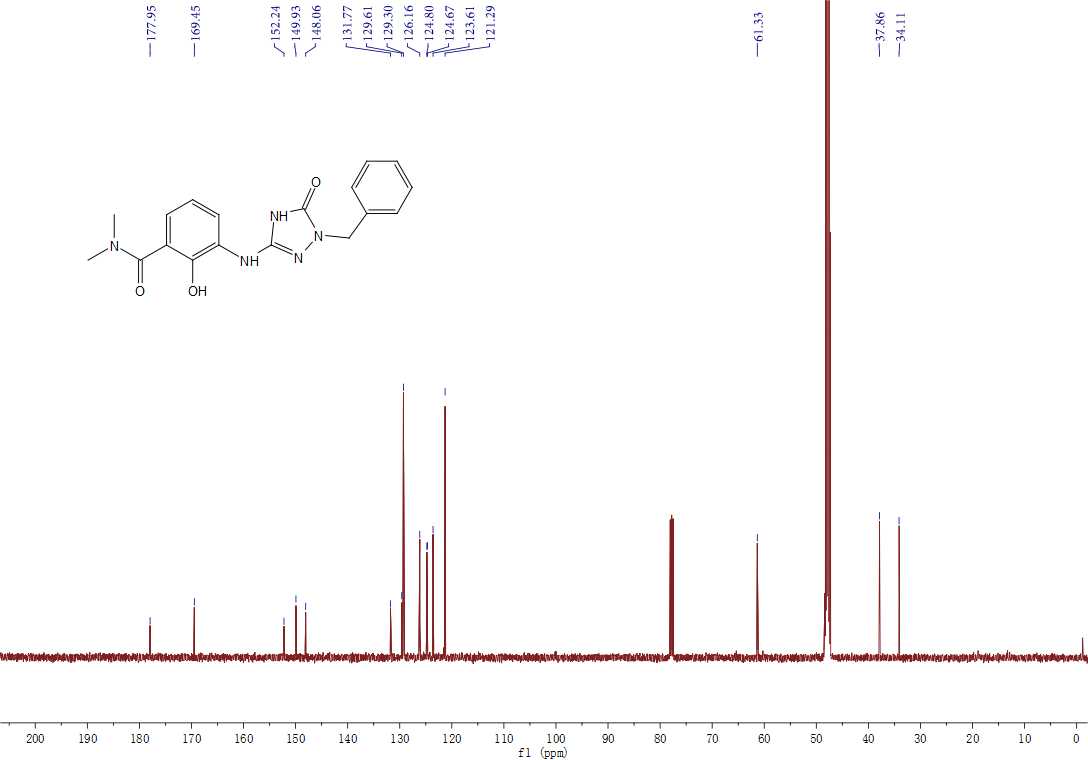


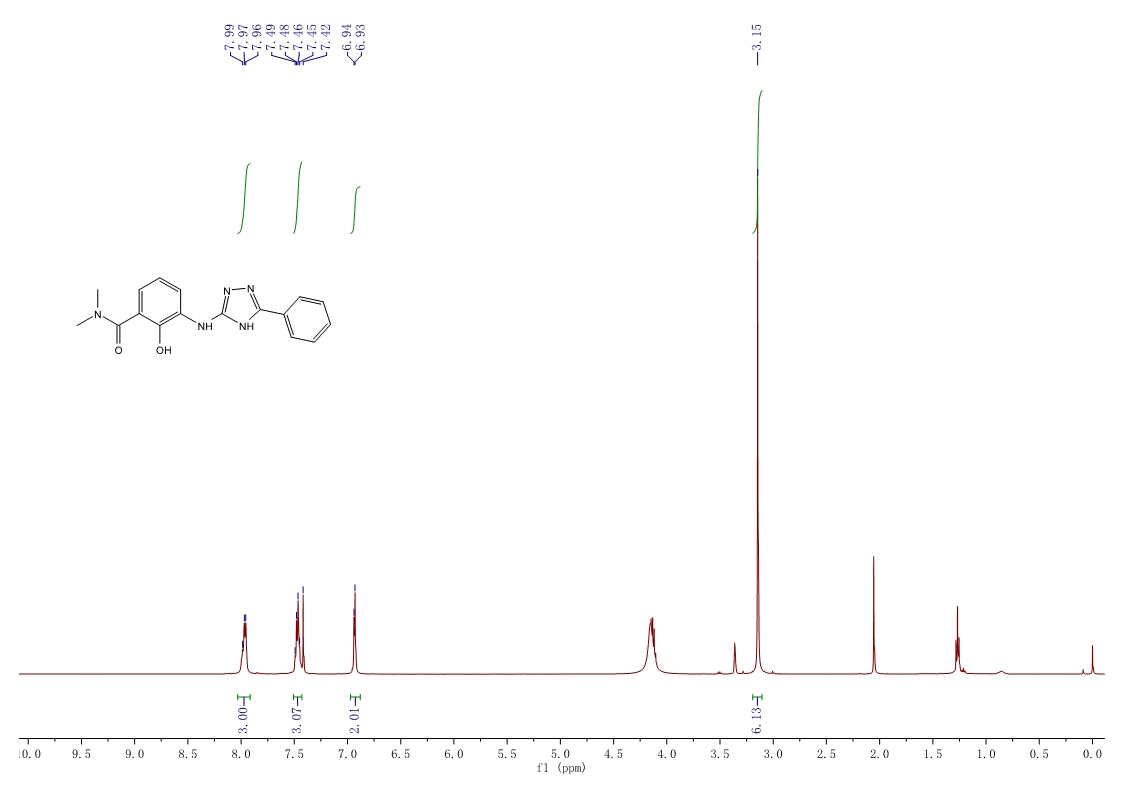


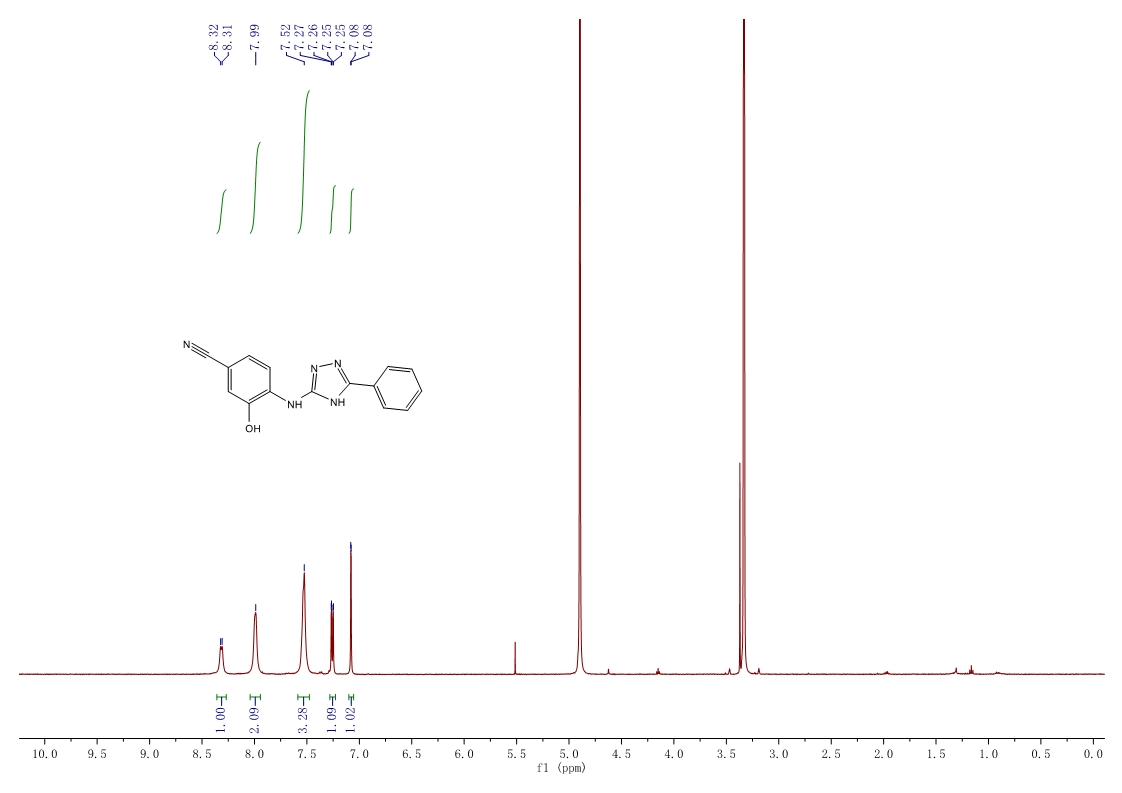


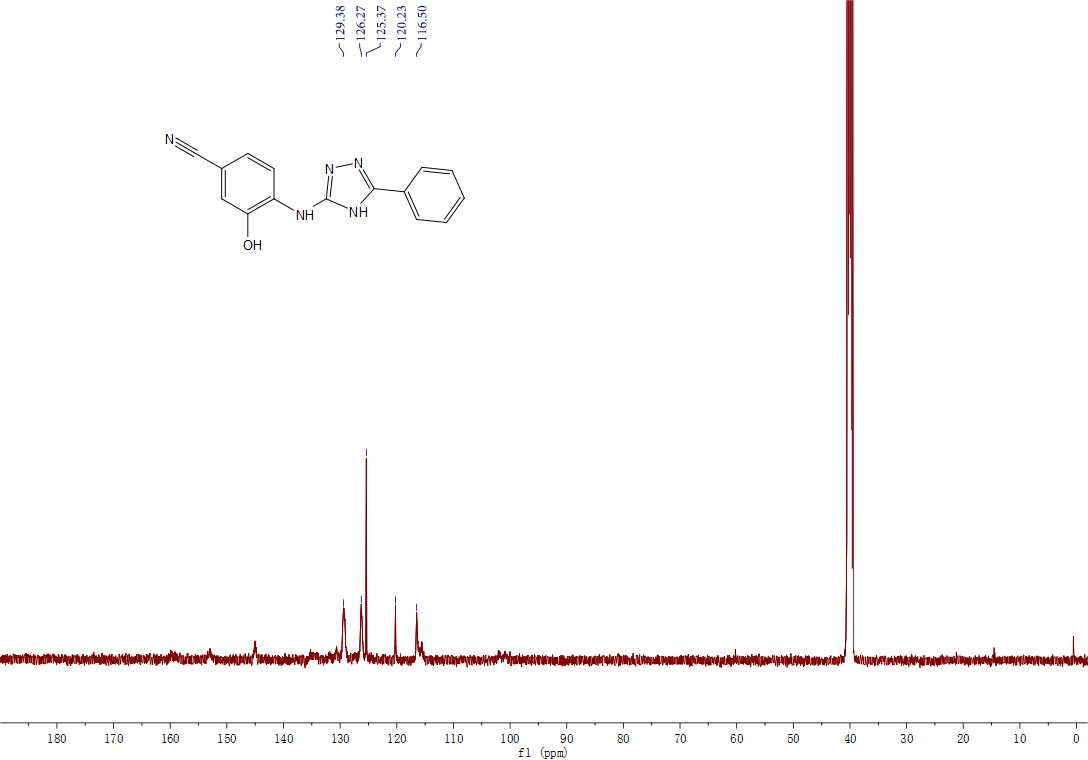


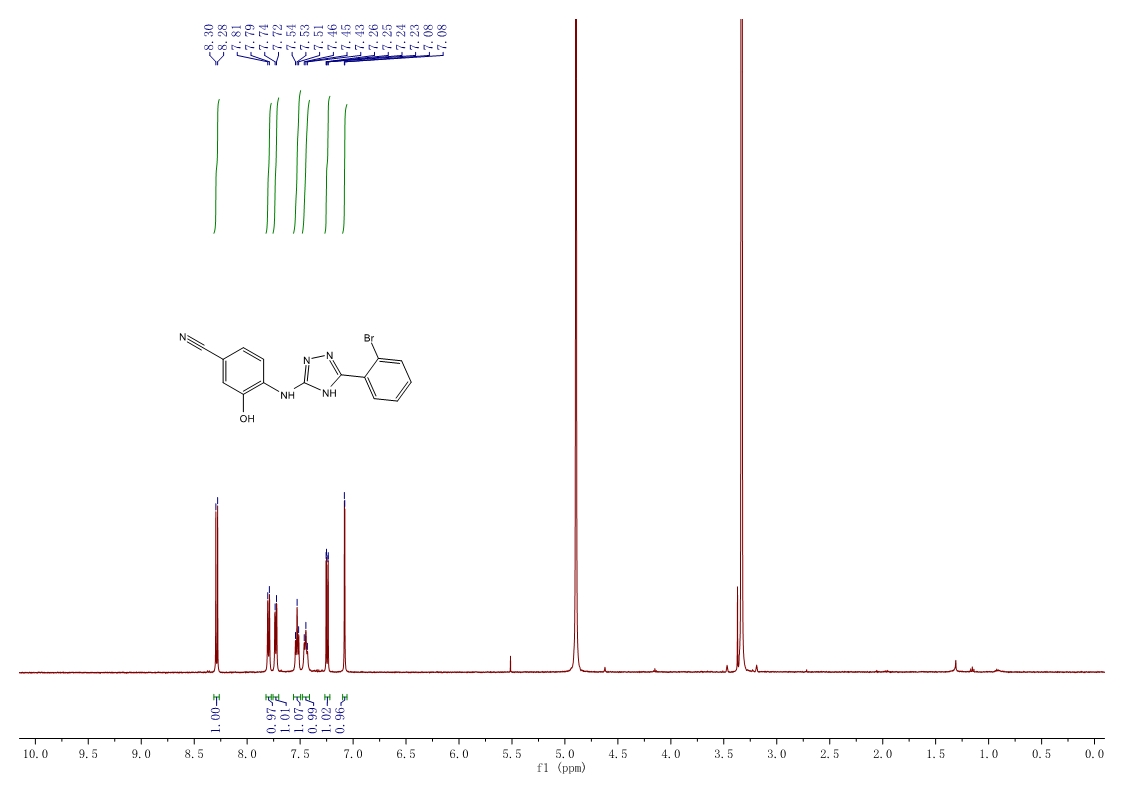


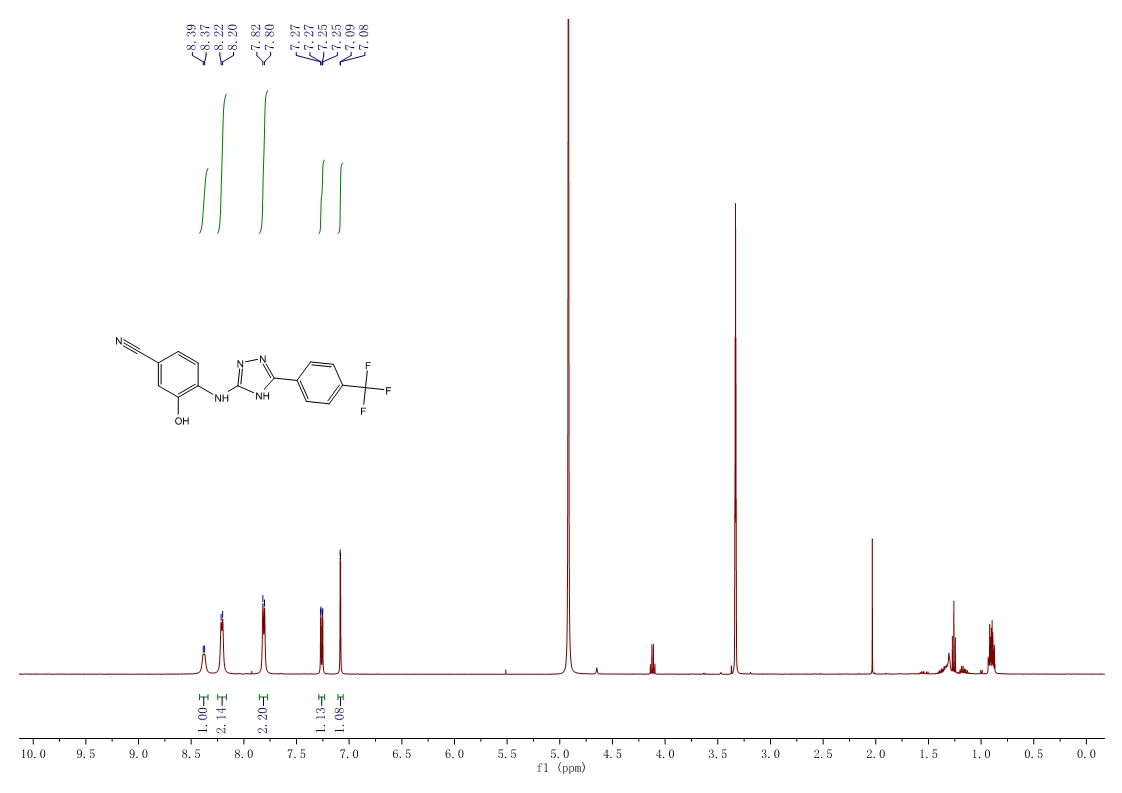


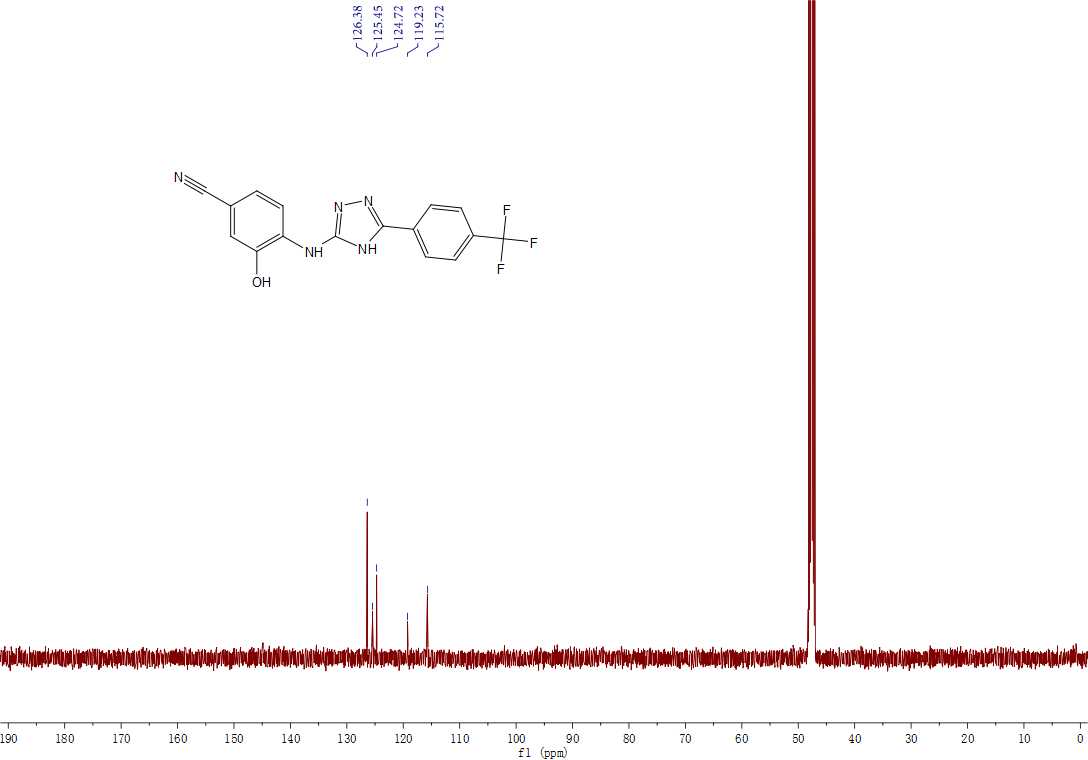


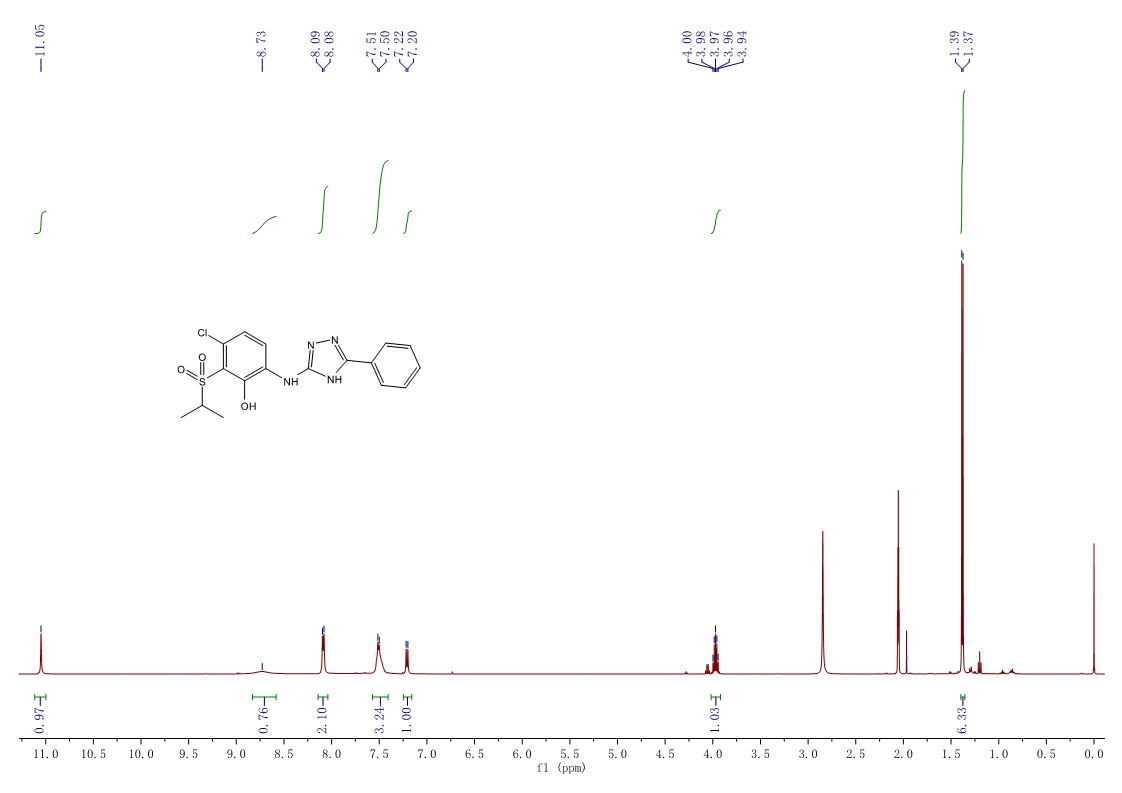


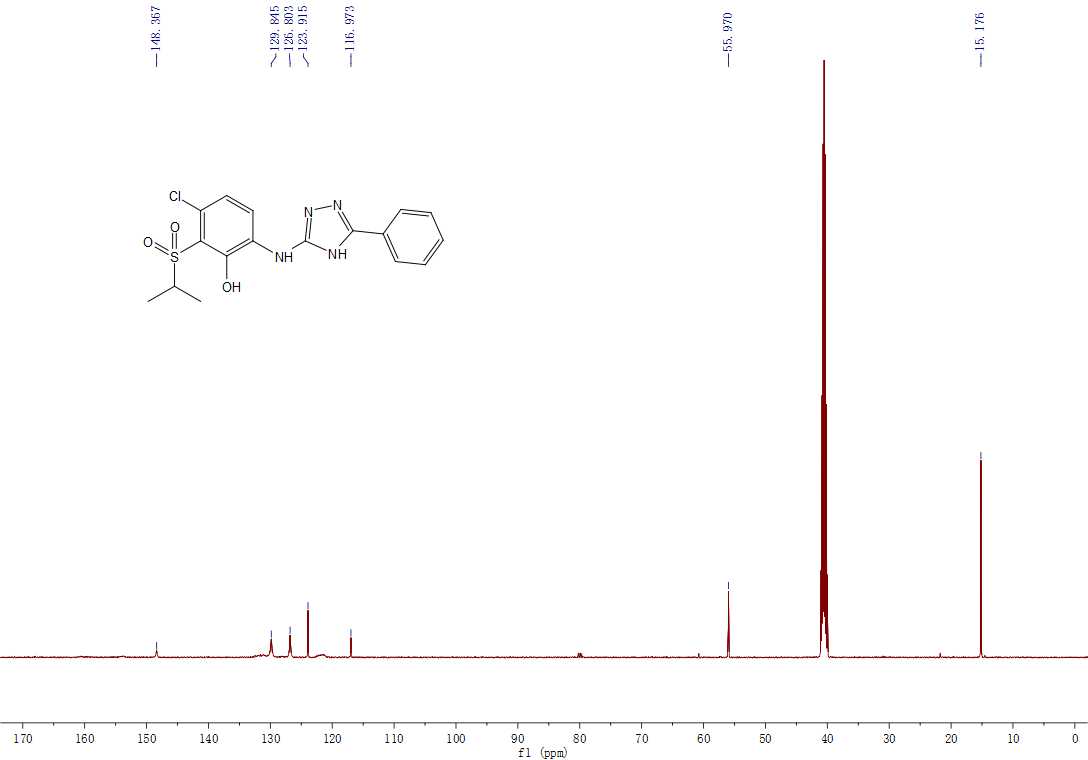

Supplement: Parameters of pharmacophore hypothesis generation; Parameters of validation and screening; Chemistry; NMR Spectrum of compounds [file rsos180176supp1.docx]
